# Automatic Outcome in Manual Dexterity Assessment Using Colour Segmentation and Nearest Neighbour Classifier

**DOI:** 10.3390/s18092876

**Published:** 2018-08-31

**Authors:** Edwin Daniel Oña, Patricia Sánchez-Herrera, Alicia Cuesta-Gómez, Santiago Martinez, Alberto Jardón, Carlos Balaguer

**Affiliations:** 1 Department of Systems Engineering and Automation, University Carlos III of Madrid, Avda. de la Universidad 30, 28911 Leganés, Spain; scasa@ing.uc3m.es (S.M.); ajardon@ing.uc3m.es (A.J.); balaguer@ing.uc3m.es (C.B.); 2 Department of Physical Therapy, Occupational Therapy, Rehabilitation and Physical Medicine, Rey Juan Carlos University, Avda. de atenas s/n, 28922 Alcorcón, Spain; patricia.sanchezherrera1@urjc.es (P.S.-H.); alicia.cuesta@urjc.es (A.C.-G.)

**Keywords:** colour segmentation, CIELab, automatic counting, NN-based classifier, manual dexterity, assessment, neurological rehabilitation

## Abstract

Objective assessment of motor function is an important component to evaluating the effectiveness of a rehabilitation process. Such assessments are carried out by clinicians using traditional tests and scales. The Box and Blocks Test (BBT) is one such scale, focusing on manual dexterity evaluation. The score is the maximum number of cubes that a person is able to displace during a time window. In a previous paper, an automated version of the Box and Blocks Test using a Microsoft Kinect sensor was presented, and referred to as the Automated Box and Blocks Test (ABBT). In this paper, the feasibility of ABBT as an automated tool for manual dexterity assessment is discussed. An algorithm, based on image segmentation in CIELab colour space and the Nearest Neighbour (NN) rule, was developed to improve the reliability of automatic cube counting. A pilot study was conducted to assess the hand motor function in people with Parkinson’s disease (PD). Three functional assessments were carried out. The success rate in automatic cube counting was studied by comparing the manual (BBT) and the automatic (ABBT) methods. The additional information provided by the ABBT was analysed to discuss its clinical significance. The results show a high correlation between manual (BBT) and automatic (ABBT) scoring. The lowest average success rate in cube counting for ABBT was 92%. Additionally, the ABBT acquires extra information from the cubes’ displacement, such as the average velocity and the time instants in which the cube was detected. The analysis of this information can be related to indicators of health status (coordination and dexterity). The results showed that the ABBT is a useful tool for automating the assessment of unilateral gross manual dexterity, and provides additional information about the user’s performance.

## 1. Introduction

Neurological disorders are diseases of the central and peripheral nervous system [1]. It involves damage of the brain, spinal cord, cranial nerves, peripheral nerves, nerve roots, autonomic nervous system, neuromuscular junction, and muscles. These disorders include epilepsy, Alzheimer disease, cerebrovascular diseases including stroke, multiple sclerosis, Parkinson’s disease, among others. The measurement of motor function is critical to the assessment and management of neurological diseases. This assessment has the potential to provide a glimpse of the patient’s clinical state beyond the consultation [2]. Clinical measures that quantify upper limb (UL) function are needed for the accurate evaluation of patients and to plan intervention strategies [3].

The functional assessment is commonly performed by health professionals themselves, using standardized scales for the sake of an objective evaluation. One of the most commonly used scales in clinical trials is the Fugl Meyer Assessment (FMA) test [4]. FMA is designed to assess motor functioning, balance, sensation and joint functioning of both upper and lower extremities [5]. Focusing on the upper limbs assessment, the Wolf Motor Function Test (WMFT) or the Action Research Arm Test (ARAT) can be found as the most common performance-based outcomes measures in stroke rehabilitation [4]. The Box and Blocks Test (BBT) [6] or the Nine Hole Peg Test (NHPT) are categorized as appropriate outcome measures in manual dexterity assessment.

Despite the previously mentioned scales intending to be objective, they could be influenced by the subjectivity of the observer [7]. In this regard, advances in technology could allow for the objective assessment of motor performance and may be used to explore motor impairments in neurological rehabilitation [8,9]. The development of objective and quantitative rehabilitation treatment assessment methods to address that issue is a non-trivial problem [10].

On this basis, several works can be found focusing on automation of the previously presented scales for upper limb functional assessment. For example, a framework for automating upper-limb motor assessments that uses low-cost sensors to collect movement data is presented in [11]. This framework is used to automate the 73% of the upper limb portion of the FMA. A recent study for FMA automation is presented in [12] that uses Kinect v2 (Microsoft Corp., Redmond, WA, USA) and force sensing resistor sensors owing to their convenient installation as compared with body-worn sensors. An automated system based on the WMFT is proposed in [13], using wearable sensors to measure the time spent in completion on 7 of the 17 test items. The sub-set 4 of ARAT is considered for automation in [14], using a sensorized cube of 7.5-cm size. Additionally, a study to automate the Timed Up and Go (TUG) test in order to discriminate low vs. high risk of fall individuals as objectively as possible using several quantitative parameters is presented in [15].

Regarding manual dexterity assessment, a digital version of the Box and Blocks Test (DBBT) is presented in [16]. Such work proposes an algorithm for automatic cube counting by using a Kinect V1 sensor. The success rate in cube counting is of 100% until 20 cubes. In addition, the hand movements are tracked. In one of the most recent works [17], using the Kinect V2 and computer vision is presented. In this case, the success rate in cube counting is 100% until 30 cubes. In addition, the automatic test administration was addressed by means of a graphical interface. These kinect-based systems offer a non-invasive method to hand motion tracking and data acquisition, in contrast with the use of systems based on wearable sensors such as a glove [18] or inertial measurement units [10,19]. It can be noted that a common goal is to obtain automatic evaluation platforms that are objective, that have repeatability, and can provide additional information than the one obtained with the traditional scales.

In this paper, a feasibility study of a Kinect-based system to automatically obtain the score of the BBT for gross manual dexterity assessment is presented. The remainder of this paper is organized as follows: Section 2 presents the material used and the design considerations for system implementation. Section 3 explains the process to automatically obtain the number of cubes, based on image segmentation in the CIELab colour space and the pixels classification using the Nearest Neighbour (NN) rule. Section 4 describes the pilot trial conducted to evaluate the proposed system with real users. Three sessions for assessing the motor function of the hand, with nine participants with Parkinson’s disease, were carried out. The methods used for usability and statistical analysis are also shown. Section 5 summarizes the results of the pilot trial. The objective data obtained with the proposed system are presented, including the effectiveness in cube counting. The reliability of the ABBT is studied, by analysing the correlation between the manual counting (BBT) versus the automatic one (ABBT). Section 6 discusses the performance and feasibility of the proposed system as a clinical tool. Finally, concluding remarks are presented in Section 7.

## 2. Material and Methods

### 2.1. The Box and Blocks Test (BBT)

**Figure 1:**
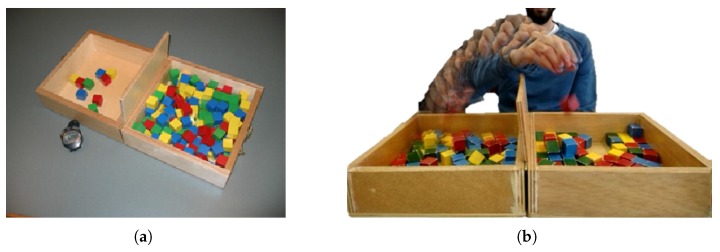
Box and Blocks Test. (**a**) Components: wooden box, coloured cubes and stopwatch; (**b**) user during test development.

The BBT is a clinically validated system for the individual measure of gross manual dexterity and coordination. The BBT can be used with a wide range of patients, including those with hand function deficits from neurological diseases. The test consists of a wooden box with two 290 mm side length square compartments, and 150 wooden cube-shaped blocks of 25 mm. A 100-mm high partition located between the two compartments must be overcome with the user’s hand to count the block as valid. In Figure 1a, the structure of the box used for the test is shown.

The goal of the test is to transport the maximum number of cubes from one compartment to the other in one minute, as it is shown in Figure 1b. For the score, the therapist must manually count the number of cubes transported. The development of the test includes three stages: a 15-s trial prior to testing, the procedure done with the dominant hand in one minute, and the procedure executed with the non-dominant hand in one minute. When testing begins, the subject should grasp one block at a time, transport the block over the partition, and release it into the opposite compartment to score. The blocks that are thrown from one compartment to the other must be penalized. If the subject transports two or more blocks at the same time, this has to be noted and the number subtracted from the total.

For the test administration, the wooden box with 150 blocks should be placed lengthwise along the edge of a table of standard height and the subject should be seated on a standard height chair facing the box, with the patient’s hands on the sides of the box. The examiner faces the subject and reads the instructions before the test begins. The rules and the instructions for the examiner and the subject are available in [6,20].

### 2.2. The Automated Box and Blocks Test (ABBT)

In a previous work [17], an automated system for evaluating gross manual dexterity, based on the BBT, was presented. It was referred to as the Automated Box and Blocks Test (ABBT). The automation of the test scoring was implemented by means of a counting algorithm based on colour segmentation in the RGB colour space. Additionally, the automatic administration of the test was addressed by using a graphical interface to guide the user during the performance of the test. The instructions for the test were given to the user by voice messages, in the same way as they would be provided by a clinician.

**Figure 2:**
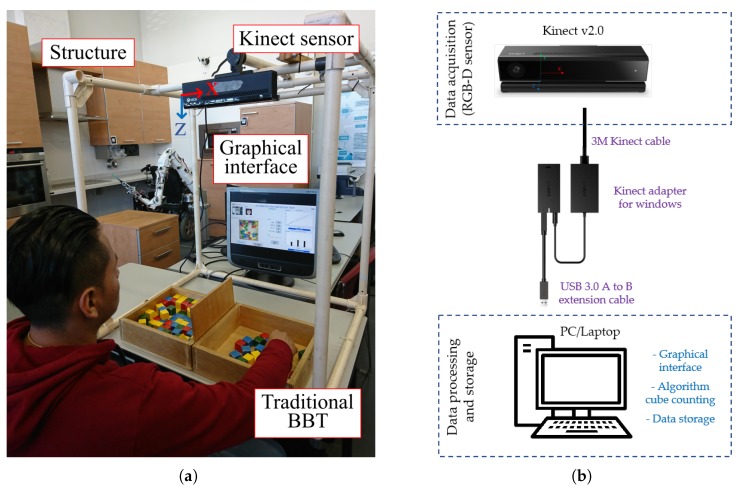
The Automated Box and Blocks Test. (**a**) Components: Kinect sensor (at the top of the portable structure), the BBT box, and the graphical Interface to allow the automatic test administration; (**b**) hardware connection.

The components of the ABBT are shown in Figure 2a. The system is made up by: (a) a portable and lightweight cube-shaped structure; (b) a Kinect for Windows V2 sensor; (c) a graphical interface (display) running on a laptop where the system also executes the counting algorithm; and (d) a traditional BBT box. The hardware architecture is depicted in Figure 2b.

To perform the test, the support structure is placed on a standard desk. At the top of the structure, the Kinect sensor is fixed with the *z*-axis of the sensor pointing to the desk. This is used for detecting the number of cubes displaced, as well as the hand movements while the subject performs the test. The BBT box is located on the desk and in the centre of the structure. In addition, a display is placed on the desk to guide the user and to show the results. The user must be seated in front of the BBT box, in the same manner as when conducting the test in the traditional way.

#### Problem Statement

The BBT is a clinically validated tool for assessing the unilateral gross manual dexterity and coordination, commonly used in neurological rehabilitation. However, the test administration is time-consuming and labour intensive. In addition, the outcome is obtained by manual counting of the transferred cubes, which could lead to an error in the measurement and it does not provide additional information about the user performance.

The ABBT, an automated version of the BBT, was previously used in a pilot study to measure the gross manual dexterity in people after a stroke [17]. This study showed that the effectiveness of the cube counting by using colour segmentation in RGB colour space was decreased by the influence of the ambient light conditions and the higher displacement speed of the cubes when they are transferred with the dominant hand.

For that reason, in the present paper, the need to improve the effectiveness of the algorithm for cube counting is addressed. Taking into account the high contrast between the colours of the cubes (red, green, blue and yellow) and also with the background of the box (beech colour), the CIE L*a*b* colour space [21], also referred to as CIELab, is employed so as to improve the success rate in automatic cube counting.

Furthermore, one of the design principles for the proposed system was to not alter the physical structure of the BBT box and the cubes. That is, using a white background or changing illumination conditions are not allowed. Thus, the effects of the reflective surfaces must be reduced, taking into account that the finish of the BBT box and the cubes is lacquered and glossy, respectively. The properties of the CIELab colour space are also appropriated to solve that issue, since the lightness channel is independent of the colour channels. This property also helps by nature to reduce the sensitivity to changes in ambient light.

## 3. Automatic Process for Cube Counting

The automatic procedure for cube counting is developed in three steps. The first step is a procedure to identify the empty compartment of the BBT box. It should be noted that it must always be an empty compartment at the beginning of the test. The cubes will be transferred to this compartment. This compartment could be the right or the left one, according to the hand to be evaluated (dominant or non-dominant hand). In order to start the test, the algorithm looks for the edges of the box, using the depth data of the Kinect sensor. Consequently, both the left and the right compartments of the box are identified. Based on this, a region of interest (ROI) in the colour image is extracted to be processed.

The second step is to run the algorithm for cube counting. This algorithm is based on colour segmentation in the CIELab colour space and the Nearest Neighbour (NN) rule. Until the period of time for the test is over, the counting process is executed.

For the third step, the validity in cubes displacement is checked since it is only allowed to displace one cube at a time. When the period of time of the test is over, the results are displayed through the graphical interface.

This three-step sequence is used for each test stage. This method, combined with voice and text messages to give the instructions to the patient, allows the ABBT to be administered automatically. The following sections detail the steps for the automatic cube counting.

### 3.1. Compartments Identification

**Figure 3:**
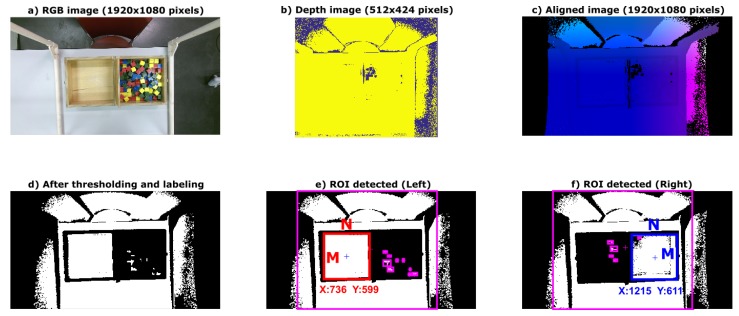
Procedure for automatic compartment identification. (**a**) Colour image (1920 × 1080 pixels); (**b**) depth image (512 × 424 pixels); (**c**) colour and depth images aligned (1920 × 1080 pixels); (**d**) image after the threshold of height is applied; (**e**) left compartment detected with the position of centroid (red line); and (**f**) right compartment detected with the position of centroid (blue line).

The procedure to detect the edge of the box and to identify the empty compartment is described in the following. The Kinect sensor is placed on the top of a structure at a vertical distance of 80-cm from the desk surface. This position remains unchanged. First, the user is asked to remove their hands from the box compartments, through a message in the graphical interface. A colour image of the scene is shown in Figure 3a. Then, depth data of the scene is captured (see Figure 3b) and a height threshold is applied. This height threshold was empirically obtained by several trials in laboratory. The distance from the Kinect sensor to the compartment edges remains unchanged, since the sensor is fixed to the structure.

It can be noted that the size of the colour image and the depth image are not the same. Thus, prior to the thresholding process, the depth and the colour images must be aligned (see Figure 3c). Since the Kinect depth camera has limited range, some pixels in the depth image do not have corresponding 3D coordinates in the colour image. However, this fact is not relevant due to the whole user workspace being covered by the camera placement.

The depth data under the threshold level are discarded, including the values corresponding to the desk and the bottom of the BBT box. Thus, the remaining image offers a clear view of both the empty and the full compartments (see Figure 3d). Morphological operations to reduce noise and to label the detected areas are applied to the thresholdized image.

Extracting features from the processed image makes it possible to detect the location of the empty compartment. A matrix with the size (M×N pixels) and the position of centroid and vertices (3D coordinates) of the rectangles containing the detected areas is obtained. Based on such features, it is possible to identify the empty compartment, and if it is the right (see Figure 3e) or the left (see Figure 3f) compartment.

The compartments’ identification is quite important because it is the basis to find the ROI in the colour image to be processed by the counting algorithm. Note that, due to the depth image’s alignment to the colour image, their pixels have corresponding 3D coordinates. That is, the coordinates of the vertices and centroid of the empty compartment in the colour and depth image are the same.

Furthermore, before the counting process starts, the system checks if the compartment is empty by means of the depth sensor. If it is not empty, the graphical interface prompts for the remaining cubes to be removed and does not start the counting process.

### 3.2. Colour Segmentation and Nearest Neighbour Classifier

The colour of an object can be described by several colour coordinate systems, and some of the most popular systems is the CIELab colour space [22]. Unlike other colour models (RGB, HSV, CMYK), the CIELab colour space correctly approximates human vision [21,23]. Similar to the RGB model, the CIELab colour space has three channels: L*, a*, and b*.

The CIELab colour axes are designed based on the fact that a colour can not be both red and green, or both blue and yellow, because these colours oppose each other. On each axis, the values run from positive to negative. On the a–a’ axis, positive values indicate the amounts of red while negative values indicate the amount of green. On the b–b’ axis, yellow is positive and blue is negative. For both axes, zero is neutral grey. The L* component represents the luminosity of the colour.

Thus, the use of the CIELab colour space is suitable for the identification of the cubes, since their colours are the same ones on which this colour space is based. Therefore, the identification of a cube’s colour only needs two colour axes (a*, b*) and the separate lightness axis (L*) is not mandatory (unlike in RGB, CMYK or HSV, where lightness depends on the relative amounts of three colour channels). This is advantageous, in that it significantly reduces the effect of changes in the environmental lighting conditions and reflective surfaces.

#### 3.2.1. Colour Markers

In automated object counting, the count is generated by capturing an image and then applying step by step image processing operations. The object counting can be done either by using a single feature or multiple features and then, by using cut-off values for those features, the final count is calculated [24]. To that end, the main features used in the present application to count the cubes are their colour, size, and shape.

First of all, it is necessary to quantify the features of the cubes in the scene. For that purpose, several experiments were performed to define some colour markers to identify the colour of the cubes inside the compartment. The average size of a single cube was also measured during experiments.

**Figure 4:**
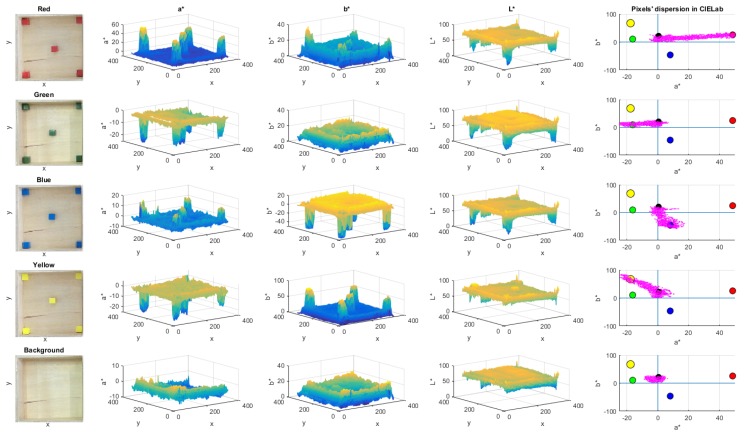
Description of the procedure to define the colour markers. The first column shows the image used for colour calibration (cubes are placed on the corners and the centre of the compartment). Columns 2 to 4 depict the a*, b*, and L* values of the scene, according to the pixel’s location (x,y). The last column describes the colour markers location and the dispersion of the CIELab colour space for each scene.

In Figure 4, the process to define the colour markers is described. The left side column shows the reference colour images taken for the calibration process. It can be noted that the colour tone is not homogeneous and it depends on the compartment zone where the cube is located, as well as environmental lighting.

For that reason, five cubes were placed in different compartment zones to define the values of colour markers. The chosen zones were the corners and the centre of the empty compartment. The three central columns in Figure 4 shows that the values of a* and b* channels of each colour are clearly different with respect to the background and other colours. In addition, the intensity levels of brightness in the L* channel is displayed.

A Canny-based edge detection is used to define the cubes’ boundaries, and to extract the colour regions. The region inside the cube frontiers is converted to CIELab colour space and its values are averaged. This procedure is repeated for each cube colour. In the case of the background, the sample positions were manually chosen from the image.

A total of 5 colour markers were defined, since the colour background of the box was included. Each colour marker has an average L*, a* and b* values. The right side column in Figure 4 depicts the location of each colour marker in the CIELab colour space, including the scatter plot of the colour pixels (denoted in magenta) for each sample image. It can be noted that the pixels are dispersed between the colour markers of the background and the corresponding colour. Thus, each pixel in the scene tends to be close to their corresponding colour marker.

#### 3.2.2. Nearest Neighbour Classifier

**Figure 5:**
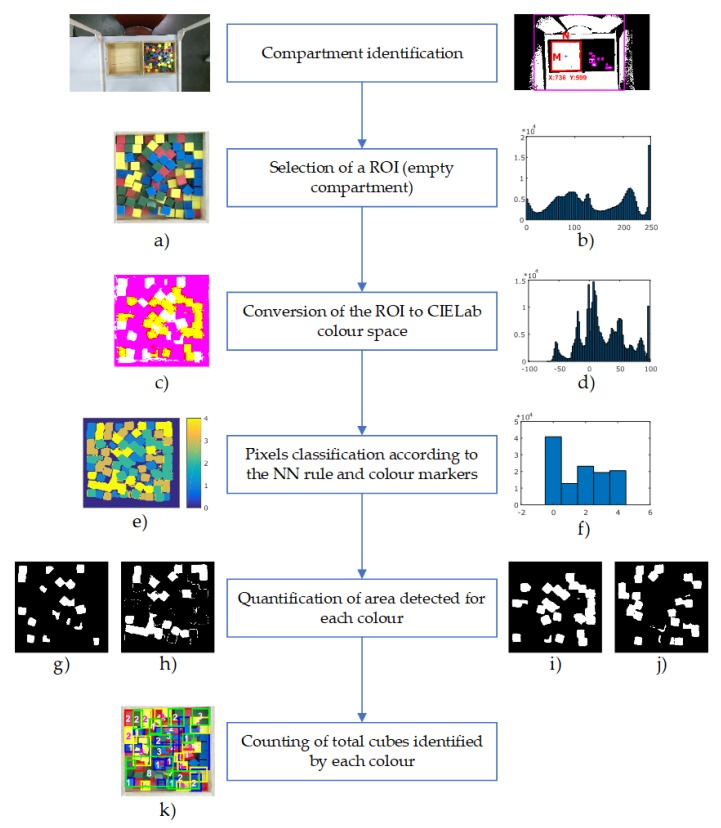
Procedure for automatic cube counting: (**a**) ROI extracted; (**b**) histogram of ROI in RGB colour space; (**c**) ROI in CIELab colour space; (**d**) histogram of ROI in CIELab colour space; (**e**) ROI labelled based on colour markers; (**f**) histogram of ROI labelled; (**g**) detected region for red colour; (**h**) detected region for blue colour; (**i**) detected region for yellow colour; (**j**) detected region for green colour; (**k**) total cube scoring for the ROI (Red: 17; Green: 22; Blue: 24; Yellow: 21).

In Figure 5, the procedure for automatic cube counting is presented. The size of the colour image captured by the Kinect sensor is 1080 × 1920 pixels in the RGB colour space.

Based on the compartment identification procedure, an ROI is extracted from the whole colour image (Figure 5a). This region corresponds to the empty side of the box (where the cubes will be transferred), and it depends on whether the test is performed for the non-dominant or dominant hand of the subject. The colour ROI is considered as an M×N matrix (see Figure 3e), where M×N is the image size in pixels. Since the compartments’ identification procedure is automated, the ROI size could be slightly different. However, the common size of this ROI is 330 × 330 pixels. The ROI is converted to the CIELab colour space so as to be processed (see Figure 5c). Then, all the pixels are classified by using the pre-calibrated values of the a* and b* channels.

The Nearest Neighbour (NN) rule is used to classify the colour pixels according to the colour markers. This algorithm assigns to a test sample the class label of its closest neighbour, based on the Euclidean distance between the sample and the class reference [25]. This technique is simple, efficient, and does not require a learning or training phase [26]. Thus, considering the colour pixels as the test sample and the colour markers as the class reference, the appropriate class label (red, green, blue, yellow or background) is assigned to each pixel in the colour image.

Euclidean distance *d* between the pixels and each colour marker is calculated by Equation (1):

(1) $$d=\sqrt{{\left({a^{*}}_{pixel}-{a^{*}}_{marker}\right)}^{2}+{\left({b^{*}}_{pixel}-{b^{*}}_{marker}\right)}^{2}}.$$

Each colour pixel is labelled according to the minimum distance to the colour markers. For example, if the distance between a pixel and the red colour marker is the smallest, then the pixel would be labelled as a red pixel. Figure 5e depicts the colour map after the classification process, where background, red, blue, yellow and green colours are labelled as 0, 1, 2, 3 and 4, respectively.

Thus, after the previous procedure, the regions of each colour (red, green, blue, yellow, and background) can be distinguished. Such regions are well defined and can be differentiated by colour labels, as is shown in Figure 5g–j. Using their colour labels, the segregated cubes are easy to detect, while in the case of cubes that are grouped, the area of each group is divided by the average area of a single cube. This average area was empirically calculated per each colour. In this way, the total amount of transferred cubes can be counted (see Figure 5k), by processing the Kinect data frame-by-frame and incrementing a global counter during the execution of the test. The classification procedure can be observed by means of the histogram of the scene. The histogram of the original scene in RGB colour space, the scene in CIELab colour space, and finally the scene after NN classification are shown in Figure 5b,d,f, respectively.

### 3.3. Score Validation

Neurological disorders cause pathophysiology of grasping due to the inability to efficiently regulate the coordination of grip and load force during object manipulation [27]. The most common deficits are paresis, ataxia, spasticity or tremor, including lack of sensitivity.

Considering such motor problems, it is possible that some individuals have trouble grabbing only a single cube, and take two at a time. In such cases, and according to the test rules [6], the additional cubes must be discarded and must be counted as one. Bearing this regulation in mind, a time vector to compare very close events is used during the performance of the test. On the basis that a healthy individual takes about a second to move a cube, it is detected whether two or more cubes have appeared in very close time instants and within a period of less than a second. In that case, the additional cubes are discarded and the global counter is only incremented by one unit.

### 3.4. Method for Automatic Test Administration

**Figure 6:**
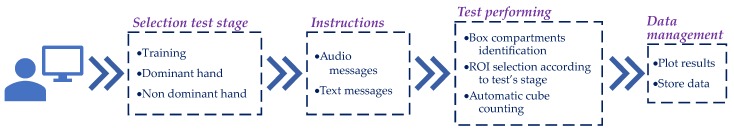
Flowchart for the test administration. The ABBT executes the appropriate sequence according to the traditional test’s rules: training, dominant hand and non-dominant hand.

The flowchart for the administration of the automated test is shown in Figure 6. The three stages of the test are administered automatically and sequentially by means of a graphical interface. First, the patient or the therapist must select the user profile, where the results of the test will be stored. In the case of a new patient, a new user profile can be created that includes the demographic data of the patient (e.g., name, age, gender, pathology, most affected side).

Once the user profile has been chosen, the test sequence is executed automatically: training, dominant hand, and non-dominant hand. At the beginning of each stage, the instructions for the test are given to the patient. Such instructions are provided by both text and voice messages through the graphical interface. Before the test starts, a dialogue box is displayed to check whether the user has understood the instructions. If not, the instructions will be given again.

At each stage of the test, the automatic procedure for counting the transferred cubes is executed. Data acquired is automatically stored in the local PC at the end of each stage.

#### Graphical Interface

**Figure 7:**
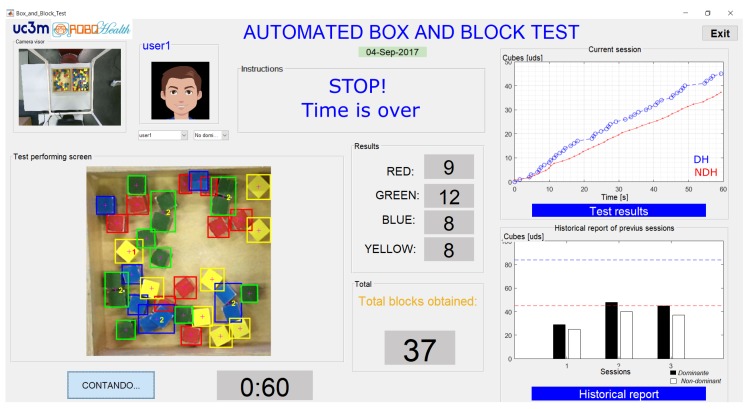
ABBT graphical interface.

The implemented graphical interface is shown in Figure 7. This is the visible part of the algorithm of cube counting and it has been designed to assist the patient during the test.

The graphical interface allows for observation of the results of the test during its execution. A full view of the Kinect colour sensor is presented in a small window (see the upper left-hand corner of Figure 7). The scoring of the cubes obtained, by colour, is also shown in the middle of the image. Furthermore, in addition to the count of the cubes by colour, there are two windows to display the results. In the first one, the cubes obtained and their times of detection for each stage are plotted (see Figure 7, upper right corner). In the second window, after the third test stage has been completed, a comparative graph of the current session along with the previous sessions is presented (see Figure 7, lower right corner), giving a historical report. Furthermore, a line that indicates the average number of cubes transferred in one minute from normative data [6] is displayed, according to the user’s demographic data.

### 3.5. ABBT Outcome

The total count of the cubes and the time instants in which they were detected are both stored for each stage of the test. This data is grouped by dominant and non-dominant hand for each subject.

On the one hand, the main outcome of the ABBT is the total amount of displaced cubes that is online calculated by the previously described algorithm. Such outcome is similar to the one provided by the traditional test.

On the other hand, therapist could be provided with additional outcome (more objective and based on user’s performance) by analysing the stored data. First, a fairly linear trend can be appreciated in the displacement of the cubes (see the top right-hand corner of Figure 7 or the figure included in Table 2) and it is related with the hand speed in transferring cubes. The linear trend can be estimated by using a simple linear regression (SLR) [17]. SLR considers only one independent variable, employing the relation $y=\beta_{0}+\beta_{1}x+\epsilon$, where $\beta_{0}$ is the *y*-intercept, $\beta_{1}$ is the slope (or regression coefficient), and ϵ is the error term.

Suppose that the set of *n* observed values of *x* and *y* is given by $\left(x_{1},y_{1}\right)$, $\left(x_{2},y_{2}\right)$, …, $\left(x_{n},y_{n}\right)$. In our case, $x_{n}$ represents a detected cube and $y_{n}$ is the time instant when it was detected. Using the SLR relation, these values yield a system of linear equations. If the line is forced to start at zero, then the system could be simplified as Y=B·X, where *B* is the slope or regression coefficient.

Then, if the variable *Y* is the register of cubes detected (NC) and the variable *X* is the time instants (*t*) when they were detected, by applying the SLR to the results obtained with ABBT, the relation that defines the ABBT outcome is:

(2) $$NC=V_{avg}\cdot t,$$

where $V_{avg}$ is the slope (*B*) from the linear fit, and it represents the average velocity in the cubes’ displacement. It is calculated for the case of dominant and non-dominant hand. The variation among the slopes can be related to the subjects’ health condition.

In addition, the partial times (PT), these being understood as the time elapsed between the displacement of one cube and the next one, are obtained from the test. Both the mean (*m*) and the dispersion (σ) of the partial times are also calculated.

## 4. Pilot Study Description

A pilot study to assess the feasibility of the automatic counting system in a real situation was conducted in a healthcare facility. A total of nine participants with Parkinson’s Disease (PD) were chosen to assess their manual dexterity. However, the participants’ symptomatology is not quite relevant to the goal of this study because the study is focused on comparing the accuracy between the manual and the automatic cube counting. Three assessment stages were carried out in different months. The first assessment was conducted in May; the second assessment in July; and the third assessment in September, all in 2017.

The ABBT settings were the same as those shown in previous Figure 2a. For each of the three assessment stages, the measurements were carried out on different days of the week. Thus, the environmental conditions were different too. As the BBT rules show, after the training period, the individuals proceed to perform the test by starting with their dominant hand (the one least affected). Then, the test was carried out with their non-dominant hand (the most affected). At the end of each test stage, one of the therapists proceeded to double count the total number of cubes displaced. This manual scoring will be compared with the automatic one.

It must be noted that the participants were attending to their regular therapy between the assessment sessions. However, the analysis of the effect of the treatment on the improvement of the health status of the participants is outside the scope of this paper.

### 4.1. Participants

Demographic data and health status of the participants in the study are summarized in Table 1. Nine individuals with PD were selected according to the following inclusion criteria: patients with PD who fulfilled the modified diagnostic criteria of the Brain Bank of the United Kingdom; patients in stages II, III and IV of the Hoehn and Yahr scale; >60% Schwab & England functionality scale; patients whose motor response to pharmacological treatment was stable or slightly fluctuating; and who were not receiving specific UL rehabilitation treatment at the time of the study.

The exclusion criteria were: the diagnosis of other diseases or serious injuries that limited occupational performance; patients presenting Parkinsonism symptoms other than PD; cognitive impairment affecting language or comprehension and the ability to follow the instructions of the study; refusal to participate in the study; stages I or V of the Hoehn and Yahr scale; and visual impairment not correctable by glasses.

### 4.2. Satisfaction Assessment

We evaluated clinicians’ perceived usability and acceptability of the ABBT by a satisfaction questionnaire. Four items are rated on a Likert-type scale from 1 to 5 (strongly disagree—strongly agree): (1) “Are you satisfied with the ABBT?”; (2) “Have the ABBT been useful in order to assess unilateral gross manual dexterity?”; (3) “Would you recommend the ABBT to other clinicians?”; (4) “Do you think that the ABBT has advantages compared to the BBT?” The arithmetic mean across all items provides the total satisfaction score. The patient’s degree of satisfaction with the ABBT was evaluated with a satisfaction questionnaire. This questionnaire consisted of a single item in which it was evaluated if the patients were very satisfied, satisfied or not at all satisfied.

### 4.3. Statistical Analysis

Since both the automated and the manual scoring are quantitative variables, Spearman’s correlation coefficient ($r_{s}$) is employed to analyse the results. The strength or magnitude of the correlation between the variables is defined based on the following criteria: negligible (correlation coefficient between 0.0 and 0.3), weak (a value between 0.3 and 0.5), moderate (a value between 0.5 and 0.7), strong (a value between 0.7 and 0.9), and very strong (a value between 0.9 and 1.0) [28]. The Shapiro–Wilk and Kolmogorov–Smirnov test were used to verify the normal distribution of the samples. The statistical analysis was performed using SPSS 24 (SPSS Inc., Chicago, IL, USA) for Windows (Windows 10, Microsoft Corp., Redmond, WA, USA).

On the one hand, to assess the reliability of the outcome obtained by using the ABBT, we performed a correlation analysis between the BBT and the ABBT scoring (manual vs. automated). In addition, the correlation between the BBT and the slopes (SLR) calculated from the single linear regression timeline dispersion obtained during the transferring of the cubes was also analysed using the same statistical method.

## 5. Results

**Figure 8:**
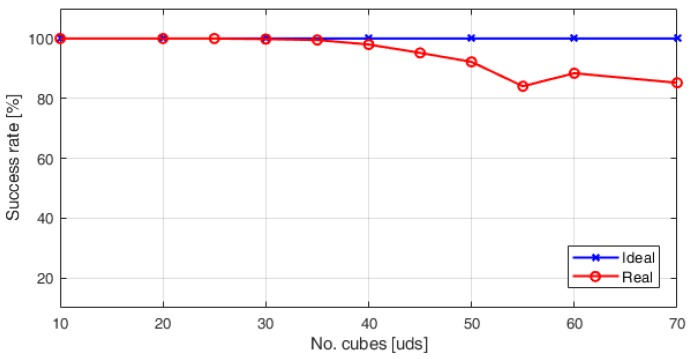
Success rate in cube counting in laboratory settings.

Figure 8 shows the number of the blocks estimated by the algorithm above described. Since the algorithm employs the segmentation by colour in CIELab colour space for cube counting estimation, it is less sensitive to changes in light conditions. After several tests, it is found that the system has 100% of accuracy when 35 blocks are transferred.

In these conditions, a pilot trial was conducted to assess the proposed system in a real situation. Several users of rehabilitation were encouraged to perform the complete test, in the same way as they perform it in a traditional assessment session, but with minimal intervention of health professionals. The system is built based on an Intel Core i7 computer (Intel, Santa Clara, CA, USA), with a Kinect for Windows V2 sensor. The implemented algorithm is able to process 3.98 images per second on average, which corresponds to a time complexity of 251 ms. The code and the graphical interface were developed in a Matlab (MATLAB R2017b, The MathWorks Inc., Natick, MA, USA) environment.

### 5.1. Data Gathered with ABBT

The data obtained by using the ABBT are summarized in Table 2, according to the assessment stages and the participants.

It can be seen that more information is obtained by using the ABBT than the one obtained with the BBT (only the number of cubes manually counted). The outcome of the ABBT is based on the analysis of the cube displacement and is made up of: the number of cubes transferred (NC), the average velocity ($V_{avg}$) in cubes displacement, and the partial times (PT). The $V_{avg}$ and PT were calculated from of previous Equation (2) and by simply subtracting the time periods that cubes were detected, respectively.

This richer set of outcomes could be useful for the clinician for improving the evaluation of the patient. A detailed comparative of the acquired data among assessment sessions for participant 1 is shown in the figure included in Table 2. It can be clearly noted that the smoothness in cubes displacement is different by comparing the plots for the dominant and non-dominant hand.

### 5.2. Analysis of the Performance of the ABBT

However, as was shown in a previous work [17], the scoring obtained by the automated method was influenced by changes in the environmental light conditions. Similarly, the hand speed in cubes displacement (different in the case of dominant and non-dominant hand) can affect to the total count. The average success ratio in such study was of 90.75% for the non-dominant hand, and of 74% for the dominant hand.

On this basis, the analysis of the performance of the ABBT with the improved algorithm was carried out. The number of cubes automatically counted by the ABBT and the manual counting of cubes (in bold) are summarized in Table 3, according to each participant and in the case of both the dominant (DH) and the non-dominant hand (NDH). The error (ϵ) in the measurement, which is understood as the fraction of $Ncubes_{-}loss/Ncubes_{-}total$ on each trial, is also presented.

The total average success ratio for automatic cube counting based on the CIELab colour space is 93.45% for the dominant hand, and 94.42% for the non-dominant hand. The error (ϵ) in the counting was calculated for each trial. The maximum errors in the measurement were 13.8% and 10.8%. However, there are no more measurements above the 10% of error in addition to those two measurements. That is, the success rate of the cube counting is above 90% in 96.3% of the performed trials (52/54).

These results prove that the algorithm for cube counting based on the CIELab colour space has a better performance than the one based on the RGB colour space, considering also that both the sample size (n=54) and the average number of cubes transferred (45.8 cubes) are larger than those studied in [17].

However, it is important to identify the causes of this error in order to still further improve the success rate of the cube counting. Figure included in Table 3 shows the histogram of lost cubes during the trials. The median number of cubes lost is 3. Between two to four cubes are lost in 70.36% of trials. The percentage of lost cubes is 48.15% and 42.59% for the dominant and non-dominant hand, respectively. Thus, the loss of cubes is equal for each hand with which the test is performed. In this sense, future developments must consider as a main source of error the occlusions caused by the layer-by-layer stacking of cubes.

### 5.3. Reliability Analysis

There were three functional assessment sessions and nine participants. Both arms were evaluated in each session. Thus, a total of 54 samples were obtained for statistical analysis.

**Figure 9:**
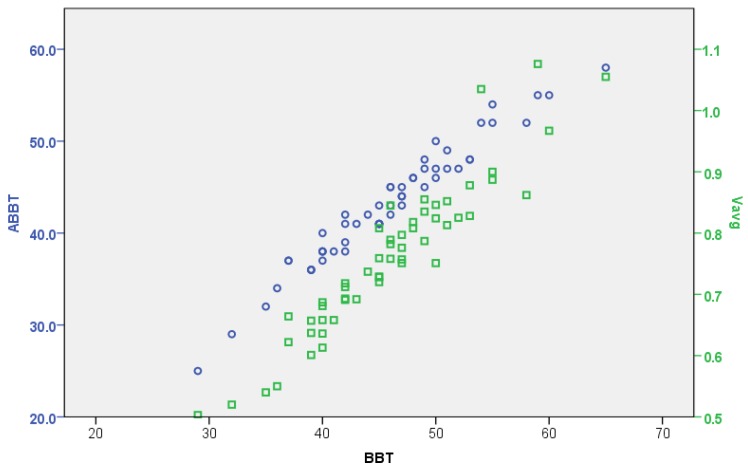
Correlation plot among test outcomes: automatic scoring (ABBT), manual counting (BBT) and average velocities ($V_{avg}$). BBT score is considered as ground-truth.

On the one hand, the reliability of the automatic (ABBT) versus the manual (BBT) counting have been statistically analysed. The global coefficient of correlation, considering the total sample (n=54), between ABBT and BBT is $r_{s}=0.98$ (Spearman’s correlation coefficient, p<0.001). On this basis, the relationship between the manual and automated scoring is found to be very strong. Figure 9 shows the previously mentioned correlation levels. Additionally, the relation between the BBT and the average velocity ($V_{avg}$) of cubes transferred has also been calculated. The coefficient of correlation is $r_{s}=0.94$, giving also a very strong level of significance.

In principle, such a global relationship could be misleading for a comparison of the performances of the dominant and the non-dominant hands. However, the coefficients of correlation between the ABBT and BBT are $r_{s}=0.967$ for the dominant hand and $r_{s}=0.978$ for the non-dominant hand. It can be appreciated that these levels of correlation are slightly lower than the global coefficient, but they are still very strong in both cases. This fact is consistent with the difference between the success ratios of cube counting for the dominant and non-dominant hand.

On the other hand, an additional analysis of the correlations between the ABBT score and another tools used for functional assessment was carried out. As it is generally done in functional assessment sessions, several tools are used for evaluation of motor function. In our case study, the clinicians measured the handgrip strength and the fine manual dexterity of participants using the Jamar dynamometer and the Purdue Pegboad Test (PPT), respectively. Such outcomes were obtained at the same time that the BBT. It was part of the regular therapy treatment of participants in the healthcare facility.

For the analysis, the hypothesis was that the relationship between the BBT and the other tools could be similar to the ABBT with the same ones. The data considered was the total sample (n=54). First, the coefficient of correlation between the gross manual dexterity (BBT) and the handgrip strength (Jamar) is $r_{s}=0.363$ (Spearman’s correlation coefficient, p<0.004). The relationship between the BBT and the PPT is $r_{s}=0.623$ (Spearman’s correlation coefficient, p<0.001). Second, the coefficient of correlation between the gross manual dexterity measured by the ABBT and the handgrip strength (Jamar) is $r_{s}=0.361$ (Spearman’s correlation coefficient, p<0.004). The relationship between the ABBT and PPT is $r_{s}=0.636$ (Spearman’s correlation coefficient, p<0.001).

**Figure 10:**
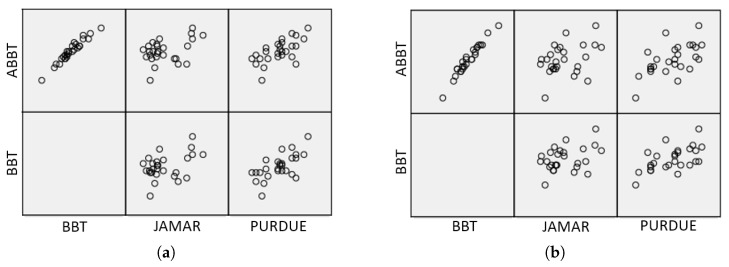
Correlation plot among three outcomes commonly used for upper limb functional assessment: (**a**) for the dominant hand, and (**b**) non-dominant hand.

It can be noted that the coefficient of correlation between the BBT and Jamar is low, and that between the BBT and PPT is moderate. Nevertheless, the same levels of correlation were obtained when using ABBT. The coefficients of correlation between outcomes are summarized in Figure 10a,b, for the dominant and non-dominant hand, respectively.

Considering the previous levels of correlation, it can be observed that the error between manual and automatic measurement is not significant. In addition, the correlations obtained between the different tools suggest that the information provided by the automatic method is as reliable as the manual one, despite of the error in the measurement.

### 5.4. Usability Assessment

The perceived usability and acceptability of the ABBT proposed here varied across clinicians. The clinicians’ answers are summarized in Table 4. The four clinicians evaluated “agreed or strongly agreed” with the satisfaction with the ABBT. Three clinicians reported that the ABBT was useful in order to assess unilateral gross manual dexterity (“strong agreement”), but the other one reported “neither agreement nor disagreement” in this regard. Regarding the degree of recommendation of the ABBT, three clinicians reported to be in “agreement or strong agreement”; however, the other participant indicated to be in “neither agreement nor disagreement”. Finally, all participants declared to “agree or strongly agree” with the advantages compared to the BBT; all the patients showed a high degree of satisfaction with the ABBT.

## 6. Discussion

In this study, the improvements to a Kinect-based system, using colour segmentation and a NN-based classifier, for automatically obtaining the score during assessment of gross manual dexterity were presented. A pilot study to evaluate the performance and feasibility of the proposed method was conducted. The viability of the proposed system was studied by including this automated method among the tools for manual dexterity assessment of patients with PD. The results obtained for each measurement were compared in order to quantify the reliability of the ABBT.

### 6.1. Comparison of the Manual and Automated Method

Correct assessment of motor function is essential for optimal rehabilitation management. There is a great need for continuous and objective monitoring of motor symptoms in neurological patients. Most of the motor scales are sufficiently validated and widely used in clinical practice. However, regarding inter-rater reliability, the motor scales have limitations [29]. The need to quickly and safely obtain accurate and reliable data is essential for clinicians to evaluate patient’s mobility status of upper extremities and determine the appropriate rehabilitation treatment. In addition, the analysis of the data gathered during the rehabilitation process is useful to detect the improvements that have occurred. The BBT is a rating scale used to measure unilateral gross manual dexterity. The test–retest reliability of the BBT is high (intraclass correlation coefficients [ICC] of 0.89 to 0.97), and the validity of the BBT has been shown by the significant correlations between the BBT and functional independence measurement [30].

On this basis, the BBT study was considered since the outcome is simple (total cubes transferred), the test instructions are systematic and clear, and the test development is well defined (three stages: training, dominant hand, and non-dominant hand) [6,31]. In addition, outside of the test design itself, it was selected for its wide use in clinical settings as an evaluation system in neurological rehabilitation [4]. The ABBT is the automated version of the BBT and provides more information than the traditional BBT. This information is stored directly in the patient’s register, easing the update of the medical history. Additionally, the cubes’ colours and the time period when they were detected are obtained and registered by the ABBT.

Regarding the performance of the automatic system of cube counting, the statistical analysis showed a very strong correlation ($r_{s}=0.98$) between the BBT and the ABBT scores. The success rate of the cube counting was improved by means of applying the CIELab colour space to detect the cubes. The results show an average success rate in the counting of cubes of 92.5% in the worst case and a maximum error of 13.8%.

In this way, the effectiveness in automatic cube counting of the proposed method, based on colour segmentation in the CIELab colour space and the Nearest Neighbour (NN) rule, has been better than previous works [16,17]. The measurement error was small and consistent during the different assessment sessions. It must be noted that the colour markers used for the NN classifier were the same during all the sessions, which were conducted in different days and months. This fact shows that the selection of the CIELab colour space is well suited for the BBT automation, since it is not significantly affected by environmental lighting conditions.

However, future developments must consider the main source of error that remains due to the nature of the application. Cubes can be stacked in different layers. This issue produces occlusions and loss of visual data. The use of depth sensors could detect this problem and the visual processing algorithm could be modified to deal with it. In addition, the use of a fuzzy logic approach is also considered for increasing the algorithm performance [32].

Regarding the clinical value of the additional data provided by ABBT, a linear relationship between the ABBT and BBT outcomes can be observed in Figure 9. In this figure, a stronger relationship between the BBT score and the displacement slopes ($V_{avg}$), given by the simple linear regression (SLR), is also depicted. However, as it was shown in the statistical analysis, the correlation level between BBT vs. SLR is lower than the BBT vs. ABBT. This can be attributed to the fact that automatic outcome is larger and more detailed than the one obtained with the BBT, since the automatic outcome not only considers the number of cubes transferred but also the movement quality. That is, given the same number of cubes transferred, the SLR can be different depending on the smoothness in the movement (level of dispersion in PT). The analysis of this information can be related to indicators of coordination or dexterity. This approach requires further trials to be refined.

### 6.2. Feasibility of the Automated Method

This study demonstrated the suitability of the ABBT to assess unilateral gross manual dexterity in an automated way. The use of the ABBT was simple, and its outcome reliable, during the functional assessment of real patients. Therefore, it would be possible to carry out this type of evaluation in clinical environments.

The clinicians were satisfied when using the tool as it would solve a primary complaint of having to be exceedingly cautious and attentive when counting the cubes to avoid possible counting errors. Thus, having mistakes when counting and consequently repeating the test could be avoided by the tool automation. Patients additionally showed high satisfaction with the ABBT. They commented that they did not find any difficulty in performing the test automatically as compared to manually, that they understood the instructions perfectly and that it was quick and easy.

Regarding the advantages of the automated method, it was highlighted by the clinicians that the possibility of having a tool like the ABBT would allow for improving the assessment by focusing attention on the patient and not on the test. For example, the physician may detect if the individual performs some type of compensation to assist in the movement, such as leaning the torso forward or forcing the shoulder. In addition, if the patient would have a problem such as fatigue or pain in the arm when performing the test, the clinician could detect it immediately due to not being aware of the cube count. In the same way, the clinician could observe the way in which the patient performs the scopes and grips of the cubes, to have a slight idea of the deficits in the upper limb. In this way, the ABBT could be a useful tool to assist the evaluator during the assessment process in clinical settings.

Additionally, it is clear that the use of ABBT in particular, and of automated systems in general, for tele-rehabilitation is promising. However, some concerns must be carefully considered. A lot of the work by the clinicians is not simply instructing or counting but keeping a check on the patient (i.e., if they are tired, bored, or need assistance). These human factors (patient encouragement, friendly interfaces, etc.) must be addressed to allow the integration and to increase the utility of such systems in tele-rehabilitation.

In conclusion, the obtained performance and the objective information provided by the proposed system, as well as the acceptance by the clinicians and patients, further support the development of automated methods for functional assessment in rehabilitation processes.

## 7. Conclusions

In this paper, an automated system using a Kinect V2 sensor for the assessment of unilateral gross manual dexterity was described. The reliability of this approach was studied as the main goal of the present article. For this purpose, the ABBT was included among the tools for assessing the upper limbs motor function of a group of patients with PD. The test was administered to nine participants in three assessment sessions. A total of 57 samples were obtained.

In this way, we proposed a hybrid method of colour segmentation and nearest neighbour classification, which deals naturally with the traditional test setting, does not need a training dataset, has reasonable computational complexity at run time, and yields excellent results in practice. In addition, since automatic counting is objective, reliable and reproducible, it improves the outcome obtained by manual counting.

In addition, taking into account the high level of correlation between the manual and the automated counting ($r_{s}=0.98$) and the additional information obtained using the ABBT, the results suggest that the proposed system can be used as a tool for the automatic assessment of manual dexterity.

The ABBT may be a promising and feasible evaluation tool for tele-rehabilitation processes, even for assessing of a group in clinical settings. This system presents important advantages like its portability, ease of use, commercial availability, inexpensiveness and non-invasive nature.

## Figures and Tables

**Table 1:** Demographics and health status of participants.

Participant	Age	Gender	Dominant Hand	Diagnosis Year
P1	72	Male	Left	2002
P2	57	Female	Left	2006
P3	54	Female	Left	2012
P4	55	Male	Left	2013
P5	45	Male	Left	2017
P6	70	Male	Left	2011
P7	65	Female	Right	2016
P8	73	Male	Left	2009
P9	71	Female	Right	2004

**Table 2:** ABBT outcome for each participant (DH: dominant hand, NDH: non-dominant hand; NC: number of cubes; $V_{avg}$: average velocity in cubes/second; PT: Average of partial times in seconds.)

(a) First Assessment				
**Participant**	**Evaluated hand**	**NC**	$V_{\mathrm{avg}}$	**PT (** m±σ **)**
P1	DH	29	0.520	2.21±0.06
	NDH	25	0.503	2.27±1.19
P2	DH	46	0.751	1.10±0.05
	NDH	41	0.729	1.14±0.02
P3	DH	45	0.845	1.19±1.17
	NDH	39	0.718	1.11±1.11
P4	DH	38	0.691	1.15±1.19
	NDH	32	0.540	1.17±0.03
P5	DH	55	1.076	1.18±1.15
	NDH	47	0.852	1.18±1.15
P6	DH	47	0.825	1.12±0.09
	NDH	41	0.759	1.14±0.04
P7	DH	41	0.808	1.13±0.08
	NDH	37	0.664	1.15±1.13
P8	DH	38	0.658	1.15±0.08
	NDH	34	0.550	1.16±1.19
P9	DH	45	0.787	1.10±0.02
	NDH	45	0.789	1.10±0.08
**Participant**	**Evaluated hand**	**NC**	$V_{\mathrm{avg}}$	**PT (** m±σ **)**
P1	DH	48	0.835	1.15±0.03
	NDH	40	0.681	1.19±0.03
P2	DH	42	0.782	1.11±0.04
	NDH	43	0.776	1.10±0.09
P3	DH	48	0.828	1.15±0.05
	NDH	41	0.712	1.16±0.02
P4	DH	48	0.878	1.13±0.05
	NDH	47	0.855	1.16±0.09
P5	DH	54	0.900	1.10±0.09
	NDH	46	0.818	1.10±0.06
P6	DH	52	0.862	1.15±0.01
	NDH	52	1.035	1.15±0.07
P7	DH	43	0.720	1.18±0.03
	NDH	38	0.687	1.16±1.17
P8	DH	41	0.692	1.14±0.09
	NDH	36	0.637	1.16±0.08
P9	DH	44	0.751	1.16±0.06
	NDH	38	0.636	1.14±0.06
(**c**) **Third Assessment**				
**Participant**	**Evaluated hand**	**NC**	$V_{\mathrm{avg}}$	**PT (** m±σ **)**
P1	DH	45	0.797	1.12±1.13
	NDH	37	0.622	1.19±0.03
P2	DH	50	0.846	1.10±0.05
	NDH	47	0.824	1.15±0.07
P3	DH	42	0.737	1.11±0.09
	NDH	37	0.613	1.11±0.01
P4	DH	52	0.887	1.13±0.06
	NDH	42	0.693	1.12±1.12
P5	DH	58	1.055	1.13±0.08
	NDH	55	0.967	1.19±0.06
P6	DH	43	0.758	1.17±0.08
	NDH	44	0.757	1.13±0.07
P7	DH	36	0.601	1.16±1.12
	NDH	36	0.657	1.18±0.01
P8	DH	41	0.728	1.15±0.05
	NDH	38	0.658	1.17±0.06
P9	DH	49	0.813	1.11±0.07
	NDH	46	0.808	1.10±0.07
(**d**) **Detail of Data for Participant 1**				
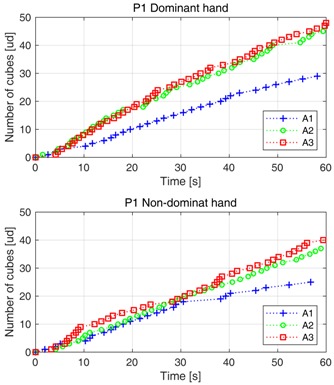				

**Table 3:** Success rate in automatic cube counting during assessment sessions. Scoring for the ABBT and the BBT (in bold) grouped by dominant hand (DH) and non-dominant hand (NDH) for each participant and for the three assessment sessions. The error in the measurement is (ϵ). Histogram of cubes loss is included.

(a) First Assessment				
**Participant**	**DH**	ϵ	**NDH**	ϵ
P1	29/**32**	0.094	25/**29**	0.038
P2	46/**50**	0.080	41/**45**	0.089
P3	45/**46**	0.022	39/**42**	0.071
P4	38/**42**	0.095	32/**35**	0.086
P5	55/**59**	0.068	47/**51**	0.078
P6	47/**52**	0.096	41/**45**	0.089
P7	41/**45**	0.089	37/**37**	0.000
P8	38/**40**	0.050	34/**36**	0.056
P9	45/**49**	0.082	45/**46**	0.022
Success rate:	92.2%		93.32%	
(**b**) **Second Assessment**				
**Participant**	**DH**	ϵ	**NDH**	ϵ
P1	48/**49**	0.020	40/**40**	0.000
P2	42/**46**	0.087	43/**47**	0.085
P3	48/**53**	0.094	41/**42**	0.024
P4	48/**53**	0.094	47/**49**	0.041
P5	54/**55**	0.018	46/**48**	0.042
P6	52/**58**	0.003	52/**54**	0.037
P7	43/**45**	0.044	38/**40**	0.050
P8	41/**43**	0.047	36/**39**	0.077
P9	44/**47**	0.064	38/**40**	0.050
Success rate:	93.34%		95.5%	
(**c**) **Third Assessment**				
**Participant**	**DH**	ϵ	**NDH**	ϵ
P1	45/**47**	0.043	37/**37**	0.000
P2	50/**50**	0.000	47/**50**	0.060
P3	42/**44**	0.045	37/**40**	0.075
P4	52/**55**	0.055	42/**42**	0.000
P5	58/**65**	0.008	55/**60**	0.083
P6	43/**46**	0.065	44/**47**	0.064
P7	36/**39**	0.077	36/**39**	0.077
P8	41/**45**	0.089	38/**41**	0.073
P9	49/**51**	0.039	46/**48**	0.042
Success rate:	94.42%		94.43%	
(**d**) **Histogram of Lost Cubes**				
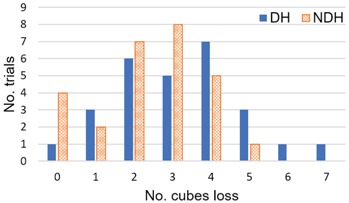				

**Table 4:** Results of satisfaction questionnaires.

Clinicians	Items	Strongly Disagree	Disagree	Neither Agreement Nor Disagreement	Agree	Strongly Agree
1	(1)					x
	(2)					x
	(3)			x		
	(4)					x
2	(1)					x
	(2)					x
	(3)				x	
	(4)					x
3	(1)					x
	(2)			x		
	(3)					x
	(4)					x
4	(1)					x
	(2)					x
	(3)				x	
	(4)					x

## Abbreviations

The following abbreviations are used in this manuscript:

ABBT	Automated Box and Blocks Test
BBT	Box and Blocks Test
CMYK	Cyan, Magenta, Yellow, Key
HSV	Hue, Saturation, Value
RGB	Red, Green, Blue
ROI	Region of Interest
SLR	Simple Linear Regression
